# The mechanical microenvironment and lung stem cell fate

**DOI:** 10.3389/fcell.2026.1836024

**Published:** 2026-05-19

**Authors:** Evelyn S. Navarro Salazar, Celeste M. Nelson

**Affiliations:** 1 Department of Chemical and Biological Engineering, Princeton University, Princeton, NJ, United States; 2 Department of Molecular Biology, Princeton University, Princeton, NJ, United States

**Keywords:** cell culture, differentiation, mechanical stress, morphodynamics, morphogenesis

## Abstract

The lung is a complex, branched organ that follows an intricate and well-coordinated developmental process to generate dozens of different cell types. Several protocols have been established to differentiate stem cells into the cell types of the lung in culture, to complement studies in animals aimed at understanding the basic processes of lung development, homeostasis, and response to injury. However, these protocols are generally both inefficient and poorly reproducible. To improve current differentiation protocols, it is important to consider how lung epithelial cells interact with their surrounding biochemical and mechanical microenvironment during development in the embryo. In this review, we describe the different microenvironmental signals that promote growth and differentiation of lung progenitors and mature lung epithelial cells. We also highlight the specific mechanical components of the microenvironment that have been recently used in cell culture models. We postulate that understanding and incorporating combinations of these microenvironmental signals to better mimic the mechanical niche can increase the efficiency, reproducibility, and physiological accuracy of culture models of the lung.

## Introduction

**FIGURE 1:**
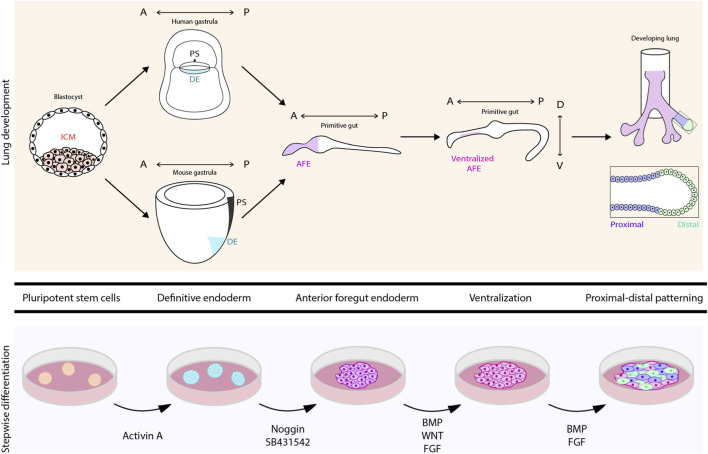
Schematic illustrating the development of mouse and human lungs from the blastocyst to the formation of the definitive endoderm (DE) during gastrulation. This DE then develops into the foregut endoderm, which patterns along the anterior-posterior axis. Ventralization of the anterior foregut endoderm (AFE) leads to the formation of lung progenitors. The lung progenitors can be further differentiated into mature lung epithelial cells found in airways and alveoli. The cell culture-based approach uses pluripotent stem cells (PSCs) obtained either from the inner cell mass of blastocysts or from the reprogramming of somatic cells. These PSCs are differentiated in a stepwise manner, following the timepoints of lung development *in vivo*.

**FIGURE 2:**
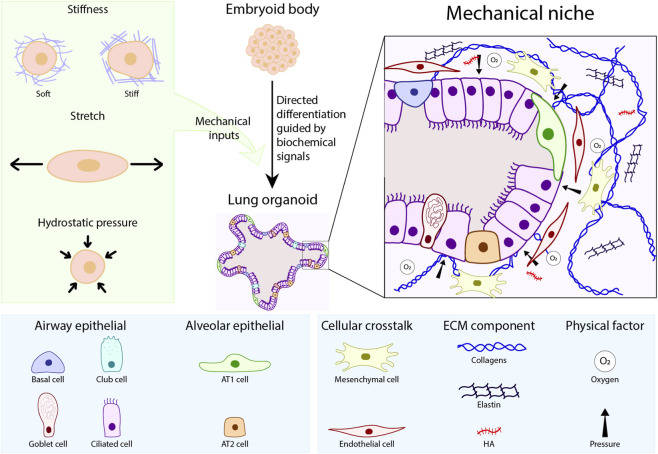
Schematic illustrating the mechanical inputs that influence cell fate decisions and organoid architecture, and the components of the “mechanical niche” (adjacent stromal cells, ECM, and other physical factors) important for the reproducibility and physiological relevance of lung models.

The lung is comprised of dozens of cell types surrounded by a variety of different extracellular matrix (ECM) proteins and characterized by region-specific mechanical properties (Uriarte et al., 2018). This critical organ arises from the anterior foregut endoderm (AFE), which itself arises from the definitive endoderm (DE) that develops soon after gastrulation. Ventral patterning of the AFE results in the specification of the lung field, which can be identified by expression of the transcription factor Nkx2-1 (Kadzik and Morrisey, 2012). After lung buds form and start branching, the lung epithelium is patterned in a proximal-to-distal manner (Figure 1). Proximal progenitors give rise to club cells, ciliated cells, goblet cells, basal cells, and neuroendocrine cells, which populate the upper airways of the lung. Distal progenitors give rise to alveolar epithelial type I (AT1) and type II (AT2) cells, which line the alveoli (Kadzik and Morrisey, 2012). Each type of progenitor is located in a distinct microenvironment characterized by the adjacent stromal cells, extracellular matrix (ECM), and other physical factors that, together, can be thought of as a ‘mechanical niche’ (Figure 2). Recent transcriptomic analyses have improved the identification of different lung cell populations, demonstrating that the human lung contains 15 epithelial cell types, which is seven more than the epithelial cell types observed in the mouse lung (Travaglini et al., 2020). This difference highlights the cellular complexity of the human lung compared to the mouse lung.

Engineering of culture models that accurately mimic *in vivo* physiological conditions is crucial for advancing research in lung development, regeneration, and diseases in humans, and complements mechanistic studies completed using rodents (Zhang et al., 2025). Cell-culture-based New Approach Methodologies (NAMs) are increasingly able to provide information on chemical hazard and risk assessment while avoiding the use of animals. However, these culture models are currently limited by issues with standardization and reproducibility, and often show differences in cell differentiation compared to their *in vivo* counterparts (Wallace et al., 2025). In this review, we describe recent work uncovering the microenvironmental signals that instruct pulmonary cell fate *in vivo*, and which might be harnessed to mimic the native mechanical niche and thereby improve reproducibility, scalability, and biological accuracy of lung stem cell differentiation in culture (Figure 2).

## Effects of cellular crosstalk

Over the past decade, a variety of protocols have been established to directly differentiate both human and mouse pluripotent stem cells (PSCs), including induced PSCs (iPSCs) and embryonic stem cells (ESCs), into lung epithelial progenitors and more mature epithelial lineages (Green et al., 2011; Longmire et al., 2012; Garreta et al., 2014; Gotoh et al., 2014; Jacob et al., 2017). These protocols typically mimic the stepwise biochemical changes within the local microenvironment that guide development of the lung epithelium *in vivo* (Figure 1). Specifically, treating PSCs with high concentrations of activin A mimics nodal signaling during gastrulation to form DE cells. These DE cells are then treated with a combination of Noggin and SB431542 to inhibit signaling downstream of bone morphogenetic protein (BMP) and transforming growth factor-beta (TGFbeta), respectively, resulting in AFE. The AFE cells are then treated with WNT, BMP, and fibroblast growth factor (FGF) to induce ventralization, giving rise to lung progenitors (Green et al., 2011). Continuing treatment with BMP and FGF results in the maturation of these lung progenitors into both proximal cell types such as club cells and distal cell types such as AT2 cells (Longmire et al., 2012; Garreta et al., 2014). Although they vary by timescale, the concentrations of additives, and dimensionality of cell culture, each established protocol has been found to give rise to some combination of lung epithelial cell types.

In addition to these biochemical signals, the embryonic lung epithelium also senses and responds to mechanical forces, highlighting the important role of the mechanical niche in lung development. Epithelial cells interact with their adjacent basement membrane through the key laminin-binding integrins α3β1, α6β1, and α6β4 (Yazlovitskaya et al., 2019), and epithelial-specific loss of α3-integrin causes defects in epithelial differentiation (Kim et al., 2009). Mechanical signaling from integrins is transmitted into alterations in cell fate indirectly via cytoskeletal tension and synergy with the Hippo-pathway effector, Yap. Indeed, loss of Yap in the lung epithelium results in gross morphogenetic defects and disruption of AT1-cell differentiation (Lin et al., 2017; van Soldt et al., 2019). In addition, the lung responds to changes in fluid volume by signaling through the stretch-activated ion channels, Piezo1 and Piezo2 (Gonçalves et al., 2023).

By contrast, our understanding of the specification and differentiation of the pulmonary mesenchyme and its role in development of the lung epithelium is limited in mice and unknown in humans. Nonetheless, recent studies have begun to reveal how this key cellular component of the mechanical niche can influence epithelial cell fate in culture. For example, lung mesenchymal progenitors can be derived from iPSCs obtained from *Tbx4* lung enhancer reporter mice by directed differentiation (Alber et al., 2023). Co-culturing these *in vitro*-differentiated lung mesenchymal cells with non-lung epithelial progenitors (Epcam+/Nkx2-1^mCherry^- cells that had not acquired a lung-specific fate by day 13 of differentiation) was found to induce lung epithelial cell fate: more than 80% of the previously Nkx2-1^mCherry^- cells express mCherry after 7 days of coculture. In addition, co-culturing these *in-vitro* differentiated lung mesenchymal cells with primary lung epithelial cells from E12.5 mouse embryos increased the yields of primary Nkx2-1^GFP^+ epithelial cells after co-culture, compared to epithelial culture alone (Alber et al., 2023). Similarly, both indirect and direct co-culture of human PSC-derived lung progenitors with irradiated human embryonic lung fibroblasts was found to promote differentiation of the former into AT2 cells in three-dimensional (3D) culture (Goltsis et al., 2024). These findings highlight the importance of mesenchymal cells within the local microenvironment in regulating lung progenitor specification and differentiation into mature lung epithelial cells.

Endothelial cells are a stromal resident important for development and disease, especially for a highly vascularized organ like the lung. Although culture models of the lung often lack integrated endothelial compartments, recent studies have successfully incorporated the endothelial component of the niche to examine their impact on lung epithelial cells. Co-culturing AT2 cells isolated from 7-9-day-old rat pups with both endothelial cells and fibroblasts promoted the differentiated phenotype of the former, including expression of surfactant proteins and formation of lamellar bodies, and improved epithelial organization into alveolar-like structures (Leiby et al., 2023). Recently, a method was developed in which endothelial cells from juvenile mice were microinjected into differentiated bronchioalveolar lung organoids with lung-resident mesenchymal cells to integrate an endothelial network surrounding the alveolar-like compartments (Ament et al., 2025). When isolated endothelial cells were included in the initial suspension of bronchioalveolar stem cells and mesenchymal cells, they were scarcely detected around the organoids that formed. These findings indicate that a more complex cellular composition that mimics the mechanical niche can improve the differentiation of lung epithelial cells and support more physiologically accurate organoid architecture, including endothelialization of alveolar-like structures.

## Effects of the ECM within the lung microenvironment

One of the key components of the mechanical niche is the ECM. The ECM composition in the lung varies across fetal, neonatal, and adult stages, as well as by location, regulating the shape, migration, and differentiation of resident cells over time (Zhou et al., 2018). The most highly abundant ECM proteins in the lung are collagens and elastin (Lang et al., 1994; Ito et al., 2019). Fetal human lung tissues contain higher levels of type I and III collagen in the pleura and the alveolar septae, as compared to adult tissues (Bateman et al., 1981). Other ECM components found in the lung include glycosaminoglycans (e.g., hyaluronic acid, HA), proteoglycans, laminin, and fibronectin (Suki and Bates, 2008; Yue, 2014; Padhi and Nain, 2020). Different developmental stages also express different isoforms of specific ECM components. For example, the combination of laminin gamma chains varies in murine embryonic and adult lung (Nguyen et al., 2005). The stiffness of the ECM in the lung is directly related to the biomechanical properties of the organ. Specifically, fibrillary ECM proteins such as collagen and fibronectin are responsible for the lung’s tensile strength, whereas elastin enables the lung’s elastic recoil (Burgess et al., 2016). The Young’s modulus of normal human adult lung tissue varies between 0.44 and 7.5 kPa, depending on the region measured, with an average of 1.96 kPa (Booth et al., 2012; Burgstaller et al., 2017). Using two-dimensional (2D) shear wave elastography, the average elasticity of human fetal lungs was observed to range from 4 to 5 kPa between 24 and 39 weeks of gestation (Nallet et al., 2022). These data suggest that the stiffness of the lung changes over the course of its development, in line with changes in the ECM composition of the niche.

Recently published work took advantage of ECM-derived natural hydrogels to mimic the biochemical and mechanical features of the niche within native lung tissues. Following 2D directed differentiation of human iPSCs into AFE, lung progenitors were encapsulated within a hybrid hydrogel combining Matrigel and HA, a key lung ECM component that is typically absent from Matrigel. This hybrid hydrogel was found to accelerate the formation and maturation of uniformly-sized organoids containing both bronchiolar and alveolar epithelial cells (Zhang et al., 2024). In a different approach, human iPSC-derived lung progenitors were cultured within Matrigel on Flexcell plates set to mimic fetal breathing movements, which generate cyclic mechanical strain on the distal niche. No differences were observed in the morphology, size, proliferation, or expression of epithelial lineage markers of the stretched organoids relative to static controls. However, more AT2-like cells were found in organoids cultured under stretch (Goltsis et al., 2024), consistent with the mechanical changes in the niche around alveoli during later stages of lung development. Matrigel, however, is derived from mouse sarcomas and has been shown to have significant batch-to-batch variability, which affects reproducibility (Aisenbrey and Murphy, 2020). To circumvent these challenges, alternative approaches that more closely resemble lung-specific ECM have been investigated. For example, hydrogels from decellularized human lungs obtained from patients with no history of lung disease retain critical components of the alveolar ECM. These hydrogels have been found to facilitate the proliferation of iPSC-derived AT2 cells and formation of alveolospheres; however, a subset of these cells transdifferentiates into AT1-like cells (Hoffman et al., 2023). In a different approach, stepwise differentiation of human ESC-derived DE cells yielded more mature lung epithelial cells, including AT2 cells and ciliated cells, on decellularized sheep lung ECM-coated plates than on fibronectin-coated plates (Noori et al., 2023). 3D culture conditions increased the yield of mature lung cells derived from the human ESC-derived DE cells. These findings highlight that incorporating the ECM composition and 3D nature of the mechanical niche can be used to enhance the directed differentiation and maturation of lung epithelial cells in culture.

The use of purely synthetic hydrogels is only beginning to be explored for creating a well-defined microenvironment and investigating the effects of mechanical stiffness on lung cell differentiation. For example, recent studies were able to propagate human iPSC-derived AT2 cells and induce their self-assembly into alveolospheres in norbornene-modified HA hydrogels free of Matrigel. However, these AT2 cells were obtained from iPSC-derived lung progenitors that were first differentiated in Matrigel (Loebel et al., 2022). More recently, human iPSCs were differentiated into lung progenitors while being cultured on engineered poly(ethylene glycol) norbornene (PEGNB) hydrogels combined with protease-degradable and cell-adhesive peptides. With this purely synthetic system, soft hydrogels (∼4 kPa) were found to produce a higher proportion of NKX2-1+ lung progenitors than stiff hydrogels (∼19 kPa) or Matrigel-coated plates (Tanneberger et al., 2025). Purely synthetic hydrogels thus allow one to mimic the stiffness of the mechanical niche in order to optimize the propagation and specification of lung progenitors.

## Effects of physical factors of the microenvironment

In addition to ECM proteins, the microenvironment of the lung is characterized by its oxygen concentration and fluid pressure, which play key roles in lung development (Gao et al., 2016; Nelson et al., 2017). The oxygen concentration in the fetal lung is lower than that of the mature lung, and gradually increases until the fetus is ready to be born to facilitate transition to breathing air (Ozan et al., 2025). During the early stages of lung development, the airway epithelium secretes fluid into the lumen, generating a positive pressure against the closed fetal larynx (Ozan et al., 2025). This fluid helps expand the lungs and provides local mechanical signals that promote lung morphogenesis and maturation (Nelson et al., 2017; Jaslove et al., 2022). Consistently, differentiating human iPSCs into mature lung organoids following a stepwise protocol in a microenvironment that mimicked the oxygen content and pressure of the lung led to transcriptional upregulation of key markers of AT1- and AT2-cell fate (Ozan et al., 2025). Increased branching and cellular distribution resembling the architecture of the distal lung were also observed.

Regardless of cell culture dimensionality, static cell cultures limit the transport of oxygen and nutrients to cells, resulting in reduced cell growth as compared to dynamic systems. For example, human iPSC-derived embryoid bodies (EBs) were differentiated into branching and budding lung organoids in large numbers in stirred bioreactors. Compared to static systems, the lung organoids harvested from bioreactor cultivation were larger. Single cell RNA-sequencing (scRNA-seq) confirmed that organoids generated by both static and stirred culture systems expressed classical markers of airway and alveolar epithelial cells, with no significant difference between them (Budeus et al., 2025). However, scRNA-seq revealed different types of mesenchymal cells and mesenchymal progenitors present in the lung organoids in static versus stirred culture, suggesting that the transport properties of the local niche might be important for maintaining these cells. Another study differentiated human iPSCs by encapsulating them in alginate hydrogel beads. The semi-permeability of alginate allowed for the diffusion of oxygen and nutrients to the encapsulated cells and the elimination of waste products. The encapsulated cells were cultured in a high-aspect-ratio vessel (HARV) rotary cell culture microgravity bioreactor that produces laminar flow, minimizing mechanical stresses on the beads while providing adequate mass transport and oxygenation (Alsobaie et al., 2023). Stepwise differentiation into AT2 cells under these conditions resulted in increased cell proliferation and faster differentiation. These results demonstrate that dynamic culture systems improve proliferation, differentiation, and cellular composition of lung organoids by mimicking the mechanical features of the local niche and overcoming the typical mass-transport limitations associated with static cultures.

## Conclusion

Several assays have been performed to determine the extent to which incorporating different aspects of the biochemical and mechanical niche affects the efficiency of differentiation protocols. Some of these include immunofluorescence and qPCR to confirm and quantify expression of lung epithelial markers like Nkx2-1 (Garreta et al., 2014), as well as transmission electron microscopy to examine the presence of lamellar bodies in differentiated AT2 cells (Gotoh et al., 2014; Jacob et al., 2017). Genetically encoded fluorescent tags fused to relevant genes such as Nkx2-1 or surfactant protein C (SFTPC), a marker of AT2 cells, have also been used to help identify and isolate the correct lineages at different steps of the differentiation process (Hawkins et al., 2017; Jacob et al., 2017). So far, approaches used to differentiate progenitors have not yielded 100% lung epithelial cells and may also contain non-epithelial lineages, such as mesodermal progenitors (Alber et al., 2023).

Time series scRNA-seq analysis of human iPSC-derived lung progenitor differentiation into AT2 cells has been performed to investigate the expression kinetics, fate trajectories, and cellular plasticity associated with PSC-directed differentiation (Hurley et al., 2020). These data were compared to developmental transcriptomic kinetics from bulk RNA sequencing of primary developing fetal and adult lung cells. This analysis revealed that only a subset of PSC-derived NKX2-1+ lung progenitors retain their cell fate as they differentiate into AT2 cells, whereas other cells give rise to non-lung endoderm cell fates. We anticipate that implementing the microenvironmental signals described above will enhance the efficiency of protocols designed to differentiate PSC-derived progenitors into the different cell types of the lung.

## References

[B1] Aisenbrey E. A. Murphy W. L. (2020). Synthetic alternatives to matrigel. Nat. Rev. Mater 5, 539–551. 10.1038/s41578-020-0199-8 32953138 PMC7500703

[B2] Alber A. B. Marquez H. A. Ma L. Kwong G. Thapa B. R. Villacorta-Martin C. (2023). Directed differentiation of mouse pluripotent stem cells into functional lung-specific mesenchyme. Nat. Commun. 14, 3488. 10.1038/s41467-023-39099-9 37311756 PMC10264380

[B3] Alsobaie S. Alsobaie T. Alshammary A. Mantalaris S. (2023). Differentiation of human induced pluripotent stem cells into functional lung alveolar epithelial cells in 3D dynamic culture. Front. Bioeng. Biotechnol. 11, 1173149. 10.3389/fbioe.2023.1173149 37388774 PMC10303808

[B4] Ament A.-L. Heiner M. Hessler M. C. Alexopoulos I. Steeg K. Gärtner U. (2025). Endothelialized bronchioalveolar lung organoids model endothelial cell responses to injury. Am. J. Respir. Cell Mol. Biol. 72, 124–132. 10.1165/rcmb.2023-0373MA 39226154

[B5] Bateman E. D. Turner-Warwick M. Adelmann-Grill B. C. (1981). Immunohistochemical study of collagen types in human foetal lung and fibrotic lung disease. Thorax 36, 645–653. 10.1136/thx.36.9.645 7031977 PMC471690

[B6] Booth A. J. Hadley R. Cornett A. M. Dreffs A. A. Matthes S. A. Tsui J. L. (2012). Acellular normal and fibrotic human lung matrices as a culture system for *in vitro* investigation. Am. J. Respir. Crit. Care Med. 186, 866–876. 10.1164/rccm.201204-0754OC 22936357 PMC3530219

[B7] Budeus B. Kroepel C. Sevindik Z. F. Buttler L. F. Klein D. (2025). Upscaling: efficient generation of human lung organoids from induced pluripotent stem cells using a stirring bioreactor. Front. Bioeng. Biotechnol. 13, 1684315. 10.3389/fbioe.2025.1684315 41404197 PMC12702919

[B8] Burgess J. K. Mauad T. Tjin G. Karlsson J. C. Westergren‐Thorsson G. (2016). The extracellular matrix – the under‐recognized element in lung disease? J. Pathol. 240, 397–409. 10.1002/path.4808 27623753 PMC5129494

[B9] Burgstaller G. Oehrle B. Gerckens M. White E. S. Schiller H. B. Eickelberg O. (2017). The instructive extracellular matrix of the lung: basic composition and alterations in chronic lung disease. Eur. Respir. J. 50. 10.1183/13993003.01805-2016 28679607

[B10] Gao Y. Cornfield D. N. Stenmark K. R. Thébaud B. Abman S. H. Raj J. U. (2016). Unique aspects of the developing lung circulation: structural development and regulation of vasomotor tone. Pulm. Circ. 6, 407–425. 10.1086/688890 27942377 PMC5130074

[B11] Garreta E. Melo E. Navajas D. Farré R. (2014). Low oxygen tension enhances the generation of lung progenitor cells from mouse embryonic and induced pluripotent stem cells. Physiol. Rep. 2, e12075. 10.14814/phy2.12075 25347858 PMC4187564

[B12] Goltsis O. Bilodeau C. Wang J. Luo D. Asgari M. Bozec L. (2024). Influence of mesenchymal and biophysical components on distal lung organoid differentiation. Stem Cell Res. Ther. 15, 273. 10.1186/s13287-024-03890-2 39218985 PMC11367854

[B13] Gonçalves A. N. Moura R. S. Correia-Pinto J. Nogueira-Silva C. (2023). Intraluminal chloride regulates lung branching morphogenesis: involvement of PIEZO1/PIEZO2. Respir. Res. 24, 42. 10.1186/s12931-023-02328-2 36740669 PMC9901166

[B14] Gotoh S. Ito I. Nagasaki T. Yamamoto Y. Konishi S. Korogi Y. (2014). Generation of alveolar epithelial spheroids *via* isolated progenitor cells from human pluripotent stem cells. Stem Cell Rep. 3, 394–403. 10.1016/j.stemcr.2014.07.005 25241738 PMC4266003

[B15] Green M. D. Chen A. Nostro M.-C. d’Souza S. L. Schaniel C. Lemischka I. R. (2011). Generation of anterior foregut endoderm from human embryonic and induced pluripotent stem cells. Nat. Biotechnol. 29, 267–272. 10.1038/nbt.1788 21358635 PMC4866999

[B16] Hawkins F. Kramer P. Jacob A. Driver I. Thomas D. C. McCauley K. B. (2017). Prospective isolation of NKX2-1–expressing human lung progenitors derived from pluripotent stem cells. J. Clin. Invest 127, 2277–2294. 10.1172/JCI89950 28463226 PMC5451263

[B17] Hoffman E. T. Uriarte J. J. Uhl F. E. Eckstrom K. Tanneberger A. E. Becker C. (2023). Human alveolar hydrogels promote morphological and transcriptional differentiation in iPSC-derived alveolar type 2 epithelial cells. Sci. Rep. 13, 12057. 10.1038/s41598-023-37685-x 37491483 PMC10368739

[B18] Hurley K. Ding J. Villacorta-Martin C. Herriges M. J. Jacob A. Vedaie M. (2020). Reconstructed single-cell fate trajectories define lineage plasticity windows during differentiation of human PSC-derived distal lung progenitors. Cell Stem Cell 26, 593–608.e8. 10.1016/j.stem.2019.12.009 32004478 PMC7469703

[B19] Ito J. T. Lourenço J. D. Righetti R. F. Tibério I. F. L. C. Prado C. M. Lopes F. D. T. Q. S. (2019). Extracellular matrix component remodeling in respiratory diseases: what has been found in clinical and experimental studies? Cells 8, 342. 10.3390/cells8040342 30979017 PMC6523091

[B20] Jacob A. Morley M. Hawkins F. McCauley K. B. Jean J. C. Heins H. (2017). Differentiation of human pluripotent stem cells into functional lung alveolar epithelial cells. Cell Stem Cell 21, 472–488.e10. 10.1016/j.stem.2017.08.014 28965766 PMC5755620

[B21] Jaslove J. M. Goodwin K. Sundarakrishnan A. Spurlin J. W. Mao S. Košmrlj A. (2022). Transmural pressure signals through retinoic acid to regulate lung branching. Development 149, dev199726. 10.1242/dev.199726 35051272 PMC8917413

[B22] Kadzik R. S. Morrisey E. E. (2012). Directing lung endoderm differentiation in pluripotent stem cells. Cell Stem Cell 10, 355–361. 10.1016/j.stem.2012.03.013 22482501 PMC3366272

[B23] Kim K. K. Wei Y. Szekeres C. Kugler M. C. Wolters P. J. Hill M. L. (2009). Epithelial cell α3β1 integrin links β-catenin and smad signaling to promote myofibroblast formation and pulmonary fibrosis. J. Clin. Invest 119, 213–224. 10.1172/JCI36940 19104148 PMC2613463

[B24] Lang M. R. Fiaux G. W. Gillooly M. Stewart J. A. Hulmes D. J. Lamb D. (1994). Collagen content of alveolar wall tissue in emphysematous and non-emphysematous lungs. Thorax 49, 319–326. 10.1136/thx.49.4.319 8202900 PMC475363

[B25] Leiby K. L. Yuan Y. Ng R. Raredon M. S. B. Adams T. S. Baevova P. (2023). Rational engineering of lung alveolar epithelium. npj Regen. Med. 8, 22. 10.1038/s41536-023-00295-2 37117221 PMC10147714

[B26] Lin C. Yao E. Zhang K. Jiang X. Croll S. Thompson-Peer K. (2017). YAP is essential for mechanical force production and epithelial cell proliferation during lung branching morphogenesis. eLife 6, e21130. 10.7554/eLife.21130 28323616 PMC5360446

[B27] Loebel C. Weiner A. I. Eiken M. K. Katzen J. B. Morley M. P. Bala V. (2022). Microstructured hydrogels to guide self-assembly and function of lung alveolospheres. Adv. Mater 34, e2202992. 10.1002/adma.202202992 35522531 PMC9283320

[B28] Longmire T. A. Ikonomou L. Hawkins F. Christodoulou C. Cao Y. Jean J. C. (2012). Efficient derivation of purified lung and thyroid progenitors from embryonic stem cells. Cell Stem Cell 10, 398–411. 10.1016/j.stem.2012.01.019 22482505 PMC3322392

[B29] Nallet C. Pazart L. Cochet C. Vidal C. Metz J.-P. Jacquet E. (2022). Prenatal quantification of human foetal lung and liver elasticities between 24 and 39 weeks of gestation using 2D shear wave elastography. Eur. Radiol. 32, 5559–5567. 10.1007/s00330-022-08654-1 35267093 PMC9279217

[B30] Nelson C. M. Gleghorn J. P. Pang M.-F. Jaslove J. M. Goodwin K. Varner V. D. (2017). Microfluidic chest cavities reveal that transmural pressure controls the rate of lung development. Development 144, 4328–4335. 10.1242/dev.154823 29084801 PMC5769635

[B31] Nguyen N. M. Kelley D. G. Schlueter J. A. Meyer M. J. Senior R. M. Miner J. H. (2005). Epithelial laminin α5 is necessary for distal epithelial cell maturation, VEGF production, and alveolization in the developing murine lung. Dev. Biol. 282, 111–125. 10.1016/j.ydbio.2005.02.031 15936333

[B32] Noori A. Mokhber Dezfouli M. R. Rajabi S. Ganji F. Ghezelayagh Z. El Agha E. (2023). Decellularized lung extracellular matrix scaffold promotes human embryonic stem cell differentiation towards alveolar progenitors. Cell J. 25, 372–382. 10.22074/CELLJ.2023.563370.1148 37434454 PMC10331443

[B33] Ozan V. B. Wang H. Akshay A. Anand D. Hibaoui Y. Feki A. (2025). Influence of microenvironmental orchestration on multicellular lung alveolar organoid development from human induced pluripotent stem cells. Stem Cell Rev. Rep. 21, 254–275. 10.1007/s12015-024-10789-1 39417930 PMC11762634

[B34] Padhi A. Nain A. S. (2020). ECM in differentiation: a review of matrix structure, composition and mechanical properties. Ann. Biomed. Eng. 48, 1071–1089. 10.1007/s10439-019-02337-7 31485876

[B35] Suki B. Bates J. H. T. (2008). Extracellular matrix mechanics in lung parenchymal diseases. Respir. Physiol. Neurobiol. 163, 33–43. 10.1016/j.resp.2008.03.015 18485836 PMC2666313

[B36] Tanneberger A. E. Blomberg R. Bilousova G. Ryan A. L. Magin C. M. (2025). Engineered hydrogel biomaterials facilitate lung progenitor cell differentiation from induced pluripotent stem cells. Am. J. Physiology-Lung Cell. Mol. Physiology 328, L379–L388. 10.1152/ajplung.00419.2024 39884665 PMC12239737

[B37] Travaglini K. J. Nabhan A. N. Penland L. Sinha R. Gillich A. Sit R. V. (2020). A molecular cell atlas of the human lung from single cell RNA sequencing. Nature 587, 619–625. 10.1038/s41586-020-2922-4 33208946 PMC7704697

[B38] Uriarte J. J. Uhl F. E. Rolandsson Enes S. E. Pouliot R. A. Weiss D. J. (2018). Lung bioengineering: advances and challenges in lung decellularization and recellularization. Curr. Opin. Organ Transpl. 23, 673–678. 10.1097/MOT.0000000000000584 30300330 PMC8669574

[B39] van Soldt B. J. Qian J. Li J. Tang N. Lu J. Cardoso W. V. (2019). Yap and its subcellular localization have distinct compartment-specific roles in the developing lung. Development 146, dev175810. 10.1242/dev.175810 30944105 PMC6526715

[B40] Wallace J. McElroy M. C. Klausner M. Corley R. Ayehunie S. (2025). Two- and three-dimensional culture systems: respiratory *in vitro* tissue models for chemical screening and risk-based decision making. Pharmaceuticals 18, 113. 10.3390/ph18010113 39861174 PMC11768377

[B41] Yazlovitskaya E. M. Viquez O. Tu T. De Arcangelis A. Georges-Labouesse E. Sonnenberg A. (2019). The laminin binding α3 and α6 integrins cooperate to promote epithelial cell adhesion and growth. Matrix Biol. 77, 101–116. 10.1016/j.matbio.2018.08.010 30193894 PMC6399080

[B42] Yue B. (2014). Biology of the extracellular matrix: an overview. J. Glaucoma 23, S20–S23. 10.1097/IJG.0000000000000108 25275899 PMC4185430

[B43] Zhang J. Marciano D. Wang L. Wang W. Gossen M. Yang M. (2024). Bioinspired hyaluronic acid-based Hydrogel fuels Bi-Directional lung organoid maturation *via* PIEZO1 and ITGB1 mediated mechanosensation. Adv. Mater. Interfaces 11, 2400194. 10.1002/admi.202400194

[B44] Zhang X. Liu H. Cheng H. Cui Y. Wang J. Yao Q. (2025). *In* vitro biomimetic models for respiratory diseases: progress in lung organoids and lung-on-a-chip. Stem Cell Res. Ther. 16, 415. 10.1186/s13287-025-04500-5 40739651 PMC12312423

[B45] Zhou Y. Horowitz J. C. Naba A. Ambalavanan N. Atabai K. Balestrini J. (2018). Extracellular matrix in lung development, homeostasis and disease. Matrix Biol. 73, 77–104. 10.1016/j.matbio.2018.03.005 29524630 PMC6129220

